# Malaria elimination gaining ground in the Asia Pacific

**DOI:** 10.1186/1475-2875-11-346

**Published:** 2012-10-18

**Authors:** Roly D Gosling, Maxine Whittaker, Cara Smith Gueye, Nancy Fullman, Mario Baquilod, Rita Kusriastuti, Richard GA Feachem

**Affiliations:** 1 The Global Health Group, University of California, San Francisco, San Francisco, CA, USA; 2 Australian Centre for International and Tropical Health, School of Population Health, The University of Queensland, Herston, Australia; 3 Department of Health, National, Center for Disease Prevention and Control, Manila, Philippines; 4 Vector Borne Disease Control Programme, Ministry of Health, Jakarta, Republic of Indonesia

## Abstract

Countries in the Asia Pacific region are making substantial progress toward eliminating malaria, but their success stories are rarely heard by a global audience. “Malaria 2012: Saving Lives in the Asia-Pacific,” a conference hosted by the Australian Government in Sydney, Australia from October 31 to November 2, 2012, will provide a unique opportunity to showcase the region’s work in driving down malaria transmission. One of the features of Malaria 2012 will be the Asia Pacific Malaria Elimination Network (APMEN), which has focused on harnessing the collective experiences of 13 countries through regional political and technical collaboration since its inception in 2009. Run by country partners, APMEN unites a range of partners – from national malaria programmes and academic institutions to global and regional policymaking bodies – to support each country’s malaria elimination goals through knowledge sharing, capacity building, operational research and advocacy.

## Commentary

**Figure 1:**
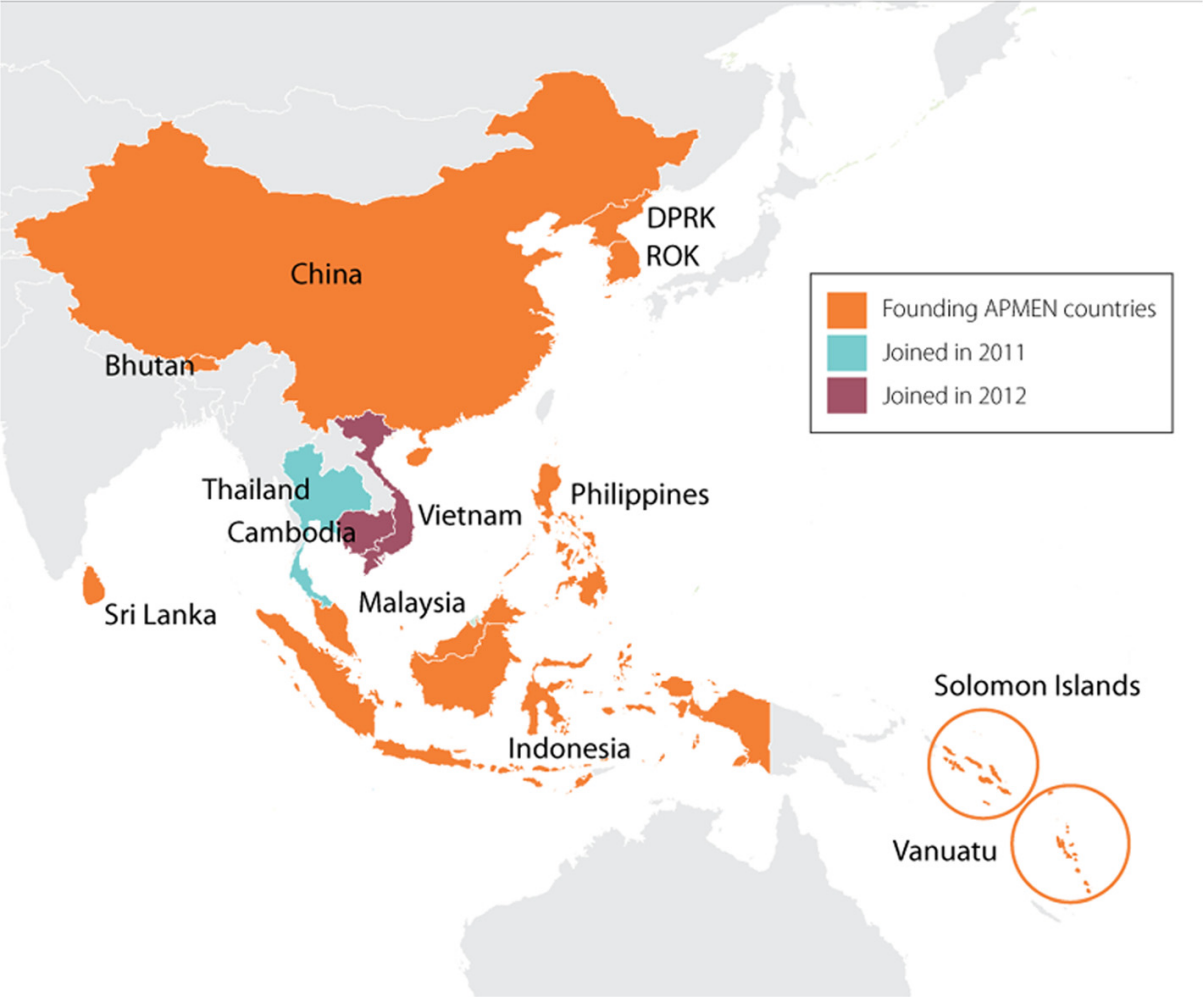
Map of the 13 countries in the Asia Pacific Malaria Elimination Network (APMEN) and when each country joined the network.

Malaria is a global disease, yet knowledge of it outside sub-Saharan Africa remains limited. As the Asia Pacific makes great strides toward eliminating malaria, its success stories are not always heard within the region, let alone among global audiences [1]. The Asia Pacific Malaria Elimination Network (APMEN) aims to fill this gap through regional political and technical collaboration, harnessing the collective experiences of 13 malaria-eliminating countries (see Figure 1) and stakeholders to form a regional initiative to foster information exchange, evidence generation and advocacy for malaria elimination.

Founded in 2009, APMEN supports malaria programme capacity building and works to elevate under-represented, region-specific issues in the global malaria arena. Substantial attention is rightfully directed towards sub-Saharan Africa and *Plasmodium falciparum* with its higher malaria morbidity and mortality [2]. Subsequently, malaria is often perceived as less problematic in places like the Asia Pacific. However, the Asia Pacific Region faces some of the most serious challenges when it comes to malaria control and elimination. The emergence of *P*. *falciparum* artemisinin drug resistance in the Mekong Region threatens local populations and, if it spreads, will have serious repercussions around the globe. Also important is the control of *Plasmodium vivax*, a relatively neglected malaria parasite [3]. This form of malaria is more widespread than *P*. *falciparum* malaria with 2.9 billion people at risk of infection, of which 90% live in the Asia Pacific [4-7].

APMEN’s Vivax Working Group addresses knowledge gaps on *P*. *vivax* through the initiative’s research grant programme, capacity-building and research collaborations. This work also aims to augment country programme capacity in research methods and implementation, including in-country technical visits. In 2012, the Working Group launched a multi-center clinical trials programme for primaquine treatment, and held workshops on genotyping *P*. *vivax* and assessing  glucose-6-phosphate dehydrogenase (G6PD) deficiency.

The Asia Pacific’s diversity of malaria vectors (19 different species) poses unique challenges for elimination because many bite and rest outside [8,9] rendering most domicile-based interventions, like insecticide-treated nets and indoor residual spraying, less effective [9,10]. To address these issues, the APMEN Vector Control Working Group collects, reviews and disseminates the latest vector control evidence and implementation strategies from the region. With its focus on building capacity, several vector control workshops are planned for 2012–2013 and the research agenda includes topics such as the effectiveness of personal protective methods for targeted high-risk groups (e.g., rubber tappers, forest workers).

To further build capacity, APMEN collaboratively designs and implements training programmes with partners, including the World Health Organization (WHO). In 2012, these focused on the use of geographic information systems for targeting resources and developing community engagement strategies for malaria elimination. Each year, APMEN provides funding for five short-term training opportunities through its Fellowship programme, allowing Fellows to learn technical skills from a partner country or institution which then can be shared with their home malaria programmes. For example, in 2010 a Fellow from Bhutan Vector-borne Disease Control Programme was hosted by the Indonesia National Malaria Control Programme to help improve strategies for community engagement.

To document programme experiences, share lessons learnt and determine priorities, APMEN hosts annual technical meetings and produces a series of country case studies that report successful approaches and challenges for malaria elimination [11,12]. During the fourth annual APMEN meeting in Seoul, held in May 2012, priorities for the next two years were set that included leveraging APMEN’s experiences to halt the spread of artemisinin-resistance by pursuing a *P*. *falciparum*-free Mekong Delta and to solve the challenges of diagnosing and radically treating *P*. *vivax*. Further learning to support these priorities comes from the cases studies. The first APMEN case study from Bhutan, demonstrated how imported malaria from neighbouring high-endemic countries affects elimination programmes [11]. Ongoing case studies in Malaysia and the Philippines focus on using intersectoral collaboration and pursuit of malaria elimination in a decentralized health system, respectively. The APMEN matrix project, an open access web-based information exchange promotes sharing of programme policies with the aim to create an evolving repository of current elimination strategies and challenges to inform future research, programme planning and capacity building in the Asia Pacific.

The most serious threat of resurgent malaria with subsequent epidemics with high mortality and drug resistance, most often caused by reductions in malaria programme financing with scaling-down of control activities, must be avoided [13]. As a unified voice advocating for malaria elimination in the region, APMEN is well positioned to galvanize the crucial political and financial support to ensure that the gains made in the fight against malaria are maintained through to elimination. This regional voice will be heard during the “Malaria 2012: Saving Lives in the Asia Pacific,” a conference hosted by the Australian Government in Sydney, Australia October 31^st^ – November 2^nd^. This summit will emphasize the importance of regional political and technical collaboration to meet control and elimination targets and to address the challenge of emerging artemisinin drug resistance. At Malaria 2012, APMEN will share its experience in unifying and supporting countries in the region fighting to be free of malaria.

## Abbreviations

APMEN: Asia pacific malaria elimination network; G6PD: Glucose-6-phosphate dehydrogenase deficiency; WHO: World health organization.

## Competing interests

RDG, MW, CSG and NF are part of the APMEN Joint-Secretariat. MB and RK serve on the APMEN Advisory Board. RGAF is the APMEN Co-Chair.
